# iPromoter-Seqvec: identifying promoters using bidirectional long short-term memory and sequence-embedded features

**DOI:** 10.1186/s12864-022-08829-6

**Published:** 2022-10-03

**Authors:** Thanh-Hoang Nguyen-Vo, Quang H. Trinh, Loc Nguyen, Phuong-Uyen Nguyen-Hoang, Susanto Rahardja, Binh P. Nguyen

**Affiliations:** 1 School of Mathematics and Statistics, Victoria University of Wellington, Gate 7, Kelburn Parade, 6140 Wellington, New Zealand; 2 School of Information and Communication Technology, Hanoi University of Science and Technology, 1 Dai Co Viet, 100000 Hanoi, Vietnam; 3 Computational Biology Center, International University - VNU HCMC, Quarter 6, Linh Trung Ward, Thu Duc District, 700000 Ho Chi Minh City, Vietnam; 4 School of Marine Science and Technology, Northwestern Polytechnical University, 127 West Youyi Road, 710072 Xi’an, China; 5 Infocomm Technology Cluster, Singapore Institute of Technology, 10 Dover Drive, 138683 Singapore, Singapore

**Keywords:** DNA, Transcription start site, Promoter, TATA-box, Bidirectional long short-term memory

## Abstract

**Background:**

Promoters, non-coding DNA sequences located at upstream regions of the transcription start site of genes/gene clusters, are essential regulatory elements for the initiation and regulation of transcriptional processes. Furthermore, identifying promoters in DNA sequences and genomes significantly contributes to discovering entire structures of genes of interest. Therefore, exploration of promoter regions is one of the most imperative topics in molecular genetics and biology. Besides experimental techniques, computational methods have been developed to predict promoters. In this study, we propose iPromoter-Seqvec – an efficient computational model to predict TATA and non-TATA promoters in human and mouse genomes using bidirectional long short-term memory neural networks in combination with sequence-embedded features extracted from input sequences. The promoter and non-promoter sequences were retrieved from the Eukaryotic Promoter database and then were refined to create four benchmark datasets.

**Results:**

The area under the receiver operating characteristic curve (AUCROC) and the area under the precision-recall curve (AUCPR) were used as two key metrics to evaluate model performance. Results on independent test sets showed that iPromoter-Seqvec outperformed other state-of-the-art methods with AUCROC values ranging from 0.85 to 0.99 and AUCPR values ranging from 0.86 to 0.99. Models predicting TATA promoters in both species had slightly higher predictive power compared to those predicting non-TATA promoters. With a novel idea of constructing artificial non-promoter sequences based on promoter sequences, our models were able to learn highly specific characteristics discriminating promoters from non-promoters to improve predictive efficiency.

**Conclusions:**

iPromoter-Seqvec is a stable and robust model for predicting both TATA and non-TATA promoters in human and mouse genomes. Our proposed method was also deployed as an online web server with a user-friendly interface to support research communities. Links to our source codes and web server are available at https://github.com/mldlproject/2022-iPromoter-Seqvec.

**Supplementary Information:**

The online version contains supplementary material available at 10.1186/s12864-022-08829-6.

## Background

Promoters are DNA non-coding regions found near and upstream the transcription start site (TSS) of genes or gene clusters [1]. As essential regulatory elements for the initiation and regulation of transcriptional processes, promoters play an important role in determining the direction and pace of DNA transcription and combining with RNA polymerase to facilitate proper initiation of transcription [2]. Understanding their molecular behaviors is also critical in investigating gene structures, assessing gene regulation methods, and annotating functional genes [3]. Besides, the initial step in explaining the transcriptional processes and expression control of genes is to map promoters to genomes [4]. Furthermore, identifying promoters in DNA sequences and genomes significantly contributes to discovering entire structures of genes of interest [5–7]. These eukaryotic transcriptional elements have typical lengths from around 60-120bp to 250bp, extending to downstream regions of the TSS [8]. For prokaryotes, the lengths of promoters extensively vary up to 1000bp [9]. Promoters may be characterized by TSS-upstream regions called TATA-boxes, which can direct other transcriptional factors to recognize the TSS [10]. The name ‘TATA-box’ comes from the nature of the region accumulating repetitive T and A base pairs (TATA). In human genomes, there are about 25% of known genes having promoters regions containing TATA-boxes [11]. In eukaryotic promoter regions, TATA-boxes are commonly ascertained at approximately 25bp upstream regions of the TSS [12]. The recognition of TATA-boxes indicates not only transcriptional directions but also which DNA strands are for binding [3]. Therefore, exploration of promoter regions is one of the most imperative topics in molecular genetics and biology.

To identify promoters, experimental techniques have been developed to improve determination efficiency and accuracy. Mutational analysis [13] and immunoprecipitation assays [14, 15] have been known as the two most prevalent used techniques. These techniques, however, are not time- and cost-effective and require skilled and experienced workers. Recently, with the extensive growth of the next-generation sequencing (NGS) technology [16], a large number of genomes have been sequenced to provide a huge source of genome data for *in silico* discovery [17–22]. This data availability has motivated researchers to develop computational models to predict promoters besides experimental approaches. So far, computational models have been developed based on signals, contents, and GpG information of sequences. Signal-based models use features extracted from information on RNA polymerase binding sites while neglecting information about neighboring sites so that their performances are usually poor [23–26]. Content-based models focus on features obtained from the calculation of *k*-mer frequencies and *k*-mer-derived features but pay less attention to the serial information of the nucleic acids in the sequence [27–29]. Unlike those two previous approaches, GpG-based models exploit locational information of GpG islands; however, GpG-based features are indistinct if just over a half of promoters possess GpG islands [30–32]. Besides, limited data sources for computational modeling was one of major limitations at that time. In recent years, science and technology have made a big leap in improving computing platforms, data storage, and computational methods to enhance computing efficiency and prediction power. Therefore, today *in silico* models have been developed with considerably elevated performances. Most of the recently developed models employ diverse types of sequence-based features [32–36]. These methods, however, mainly rely on selecting feature engineering techniques to extract sequence’s domain knowledge, and combining multiple encoding schemes may unnecessarily increase data dimensionality. Besides, developing models using traditional machine learning algorithms with high-dimensional data requires high computational costs. Deep learning, hence, can be an alternative method to construct prediction models with highly effective feature extraction integrated. Besides known successful applications in image [37], voice [38], and video [39] processing and detection, deep learning has also been widely applied in drug discovery [40], bioinformatics [41], and other scientific fields [42] to address existing shortcomings for a decade. For promoter identification, various studies have been conducted with different objectives [43–46]. In 2018, iPromoter-2L [43] was first developed for bacterial promoter prediction using random forest [47] and pseudo *K*-tuple nucleotide composition features [48]. One year after, iPromoter-2L 2.0 [44] (iPromoter-2L’s upgraded version), developed using support vector machines and *k*-mer incorporated with pseudo *K*-tuple nucleotide composition features, was released. In 2019, DeePromoter [45] was developed using convolutional neural networks, a prevalently used deep learning architecture, and one-hot encoding to predict promoters in human and mouse genomes. In the same year, Lai et al. introduced iProEP [46] for identifying promoters in multiple species, encompassing *Homo sapiens*, *Drosophila melanogaster*, *Caenorhabditis elegans*, *Bacillus subtilis*, and *Escherichia coli*, using support vector machines in combination with pseudo *K*-tuple nucleotide composition features. In 2021, Zhu et al. proposed a cross-species prediction framework called Depicter to determine three distinct types of promoters, including TATA, non-TATA, and unclassified promoters [49]. Despite satisfactory performance obtained, there is still a large room for model improvement to achieve more effective models having higher predictive efficiency, robustness, and stability.

In this study, we introduce a more effective computational model called iPromoter-Seqvec to predict TATA and non-TATA promoters in human (*Homo sapiens*) genome and mouse (*Mus musculus*) genome using bidirectional long short-term memory (Bi-LSTM) incorporated with sequence-embedded features. Long short-term memory, a deep learning architecture, belongs to a group of recurrent neural networks which are widely used in natural language processing and machine translation. For a decade, deep learning has been widely implemented to solve multiple issues in diverse fields, including biology [50], chemistry [51–53], and biochemistry [54–57]. Numerous computational approaches were developed using deep learning to address diverse biological issues [58–64]. The application of the Bi-LSTM architecture on sequence-embedded features promotes effective learning of models in forward and reverse directions with accelerated training speed compared to traditional machine learning algorithms. Sequence-embedded features, inspired by the idea of word embedding, can efficiently represent serial information of biological sequences characterized by orders of the nucleic acids in each sequence. Sequence samples used in our experiments were collected from the Eukaryotic Promoter database [65, 66] and carefully curated to create a training set, a validation set, and a test set. These datasets were controlled to be independent of each other without any repeated or highly similar sequences. To fairly assess the model performance, we compared iPromoter-Seqvec with two state-of-the-art methods: DeePromoter [45] and iProEP [46] that share common characteristics and are relevant to our study.

## Results and discussion

### Model evaluation

The model performance of iPromoter-Seqvec on the validation sets is provided in Table S1 (Supplementary Information). Since DeePromoter was also developed using ‘fake’ negative samples like ours, we reimplemented DeePromoter and evaluated its performance on the validation sets to compare the adaptivity of using ‘fake’ negative samples between iPromoter-Seqvec and DeePromoter. The results show that variation in model performance between the validation sets and the test sets for both methods is relatively small. The area under the receiver operating characteristic curve (AUCROC) and the area under the precision-recall curve (AUCPR) are two key metrics used for model evaluation. For identifying promoters in human and mouse genomes, the models predicting TATA promoters perform better than the models predicting non-TATA promoters in terms of AUCROC and AUCPR. In the aspect of other metrics, the models predicting TATA promoters for both species achieve higher values compared to those predicting non-TATA promoters. The distinct characteristics between promoters and non-promoters somehow can explain the slightly greater performance of models predicting TATA promoters in comparison with those predicting non-TATA promoters. Generally, both methods show high adaptivity to using ‘fake’ negative samples in the training model.

### Comparative analysis

Table 1 compares differences in model performance of iPromoter-Seqvec, iPro-EP, and DeePromoter. Since iPro-EP does not support predicting promoters in mouse genome, we compared the model performance based on the datasets for human genome only. To evaluate the performance of iPro-EP and DeePromoter, the test sets were uploaded to their online web servers to perform prediction tasks and retrieve predicted probabilities. For identifying promoters in human genome, iPromoter-Seqvec obtains AUCROC values of 0.99 and 0.85 for predicting TATA promoters and non-TATA promoters, respectively. The AUCPR values of iPromoter-Seqvec are also higher over those of iPro-EP and DeePromoter with 0.99 and 0.86 for predicting TATA promoters and non-TATA promoters, respectively. For identifying TATA promoters in mouse genome, AUCROC and AUCPR values of iPromoter-Seqvec are also higher than those of DeePromoter. For models predicting non-TATA promoters in mouse genome, both AUCROC and AUCPR values also confirm that iPromoter-Seqvec outperformed DeePromoter. The other metrics were also computed to provide more detailed information on model performance.

**Table 1:** Model performance on the independent test sets of iPromoter-Seqvec and other state-of-the-art methods

Dataset	Method	AUCROC	AUCPR	BA	SN	SP	PR	MCC	F1
HS-TApro	iPro-EP	0.89	0.87	0.81	0.84	0.78	0.79	0.62	0.81
	DeePromoter	-	-	0.67	0.94	0.39	0.61	0.40	0.74
	iPromoter-Seqvec (Ours)	0.99	0.99	0.94	0.90	0.99	0.99	0.89	0.94
HS-nonTApro	iPro-EP	0.73	0.74	0.65	0.73	0.56	0.63	0.30	0.67
	DeePromoter	-	-	0.51	0.90	0.12	0.51	0.04	0.65
	iPromoter-Seqvec (Ours)	0.86	0.86	0.75	0.62	0.89	0.85	0.53	0.72
MM-TApro	DeePromoter	-	-	0.59	0.84	0.34	0.56	0.21	0.67
	iPromoter-Seqvec (Ours)	0.99	0.99	0.93	0.88	0.98	0.97	0.86	0.92
MM-nonTApro	DeePromoter	-	-	0.64	0.87	0.40	0.59	0.31	0.71
	iPromoter-Seqvec (Ours)	0.91	0.91	0.83	0.74	0.91	0.90	0.67	0.81

iPromoter-Seqvec (our method), iPro-EP, and DeePromoter were developed to predict promoter regions from long DNA sequences. Also, there are other computational tools have been proposed to identify promoter sequences from limited-length DNA sequences. While prediction models like ours can answer whether any promoter region is present in DNA sequences of length up to 300bp, iPromoter-2L [43], as well as similar approaches, can only answer whether any promoter region is present in a DNA sequence of length at 81bp or lower. Nevertheless, iPromoter-2L can determine which type a promoter sequence belongs to. Hence, both approaches have their values and contributions in supporting different purposes and users.

## Conclusions

In this study, we proposed iPromoter-Seqvec, an efficient computational model using bidirectional long short-term memory neural networks and sequence-embedding features to identify TATA promoters and non-TATA promoters in human and mouse genomes. Based on evaluation metrics recorded on independent test sets, iPromoter-Seqvec is a stable and robust computational model with high AUCROC and AUCPR values. In comparison with other state-of-the-art methods, iPromoter-Seqvec shows stronger prediction power in recognizing both TATA and non-TATA promoters. Our proposed method was also deployed as an online web server with a user-friendly interface to support research communities.

## Methods

### Overview

**Fig. 1:**
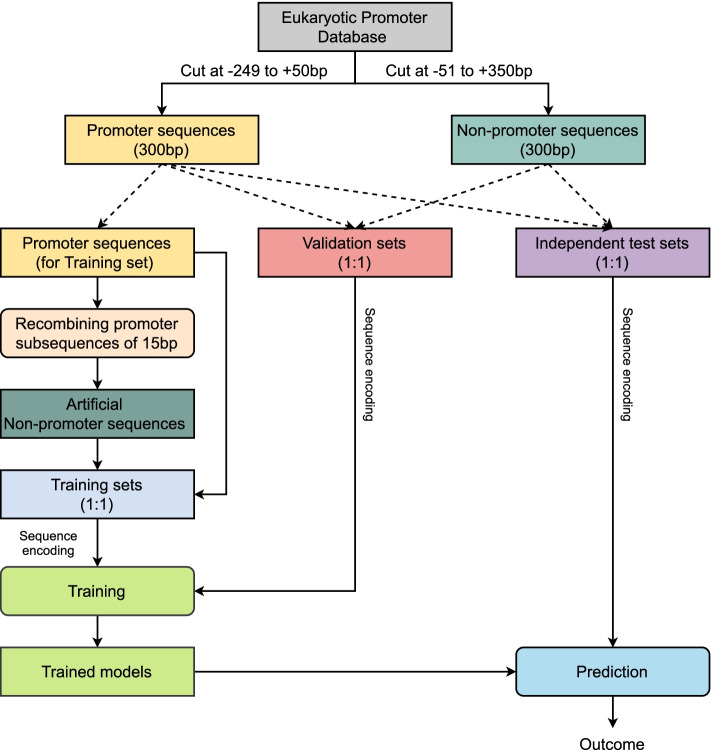
Steps in developing iPromoter-Seqvec

Figure 1 summarizes major steps in developing iPromoter-Seqvec. First, the sequence data, including experimentally verified (‘real’) promoter and non-promoter sequences, were collected from the Eukaryotic Promoter database [65, 66]. Benchmark dataset Section explains how the datasets were collected and refined. To create a validation set and an independent test set for each dataset, real promoter sequences and real non-promoter sequences were combined at an equal proportion. To create a training set, real promoter sequences were used as templates for building artificial promoter sequences. Each promoter sequence was split into smaller subsequences and then recombined to create one artificial non-promoter sequence. The detailed information on building artificial (‘fake’) non-promoter sequences is described in Construction of artificial non‑promoter sequences Section. The real promoter sequences and the fake non-promoter sequences of each dataset were combined to create a training set. The training sets were used to train models while the validation sets were used for determining at which epoch the training process should be stopped. After obtaining optimal models, the independent test sets were used to evaluate the model performance. To be recognized as the model input, all sequence data were converted to their corresponding index vectors. The index vectors stored indices of triplet sets of consecutive nucleic acids. Sequence‑embedded features Section describes the data transformation process.

### Benchmark dataset

The sequence samples used for model development and testing were collected from the Eukaryotic Promoter database [65, 66], a high-quality source of promoters. This database contains non-redundant eukaryotic POL II promoters whose TSSs have been experimentally verified. The length of all collected sequences is 300bp which were cut from a location of from -249 to +50bp (+1 refers to TSS) for promoter sequences and from -51 to +350bp for non-promoter sequences. Sequence samples were collected from data sources of both human and mouse genomes with annotated distinguishing groups: TATA promoters and non-TATA promoters. Therefore, four separate datasets, including TATA-promoters of human (HS-TApro), TATA-promoters of human (HS-nonTApro), TATA-promoters of mice (MM-TApro), and TATA-promoters of mice (MM-nonTApro) were obtained. High-similarity sequences in the four datasets were removed using the CD-HIT tool [67] with a sequence identity cut-off of 0.8. The training set of each dataset was designed with an equal number of promoter and artificial non-promoter samples. The reason and processing steps of creating artificial non-promoter samples were described in the next section. The validation and test sets of each dataset contained an equal number of promoter and non-promoter sequences. Information on datasets used for model development and evaluation is provided in Table 2.

**Table 2:** Datasets used for model training and evaluation

Dataset	No. of sequences (Promoters: Non-promoters = 1: 1)			Total
	Training	Validation	Test Set	
HS-TATApro	4958	400	500	5858
HS-nonTATApro	42800	4000	5000	51800
MM-TATApro	5272	400	500	6172
MM-nonTATApro	33892	4000	5000	42892

### Construction of artificial non-promoter sequences

In many *in silico* studies on sequence analysis, negative samples were extracted from significantly different regions. Non-promoter or non-enhancer sequences, for instance, were collected by slicing sequences from distant locations which contain non-relevant nucleic acid contents. Since the nature of positive samples (sequences of interest) and negative samples are highly distinct, models can effortlessly learn to distinguish positives from negatives. The models, therefore, can achieve very high performance but practical applications in future prediction may be limited. As promoter sequences are characterized by highly specific regions, including TATA-box (-30 to -25bp), CAAT-box (-80 to -70bp), and GC-box (-110 to -80bp), non-promoters having all these elements removed have no actual role but balancing the dataset. A large disparity between the promoters and non-promoters restricts models from learning decisive characteristics to accurately distinguish promoters from non-promoters. Models trained with bad or weak negatives find prediction tasks on genomics sequences challenging because genomic sequences enriched with promoter motifs may not be promoter sequences. The appearance of more ‘TATA’ motifs along with the genome sequences can confuse models and cause misclassifications. Hence, to develop a stable and robust model, negatives should be rigorously chosen because their features will be learned by the model to decide which class should be assigned for an unknown sample. In 2014, Wei et al. have proved the influence of good negatives on classification tasks in their studies [68]. Oubounyt et al. applied Wei et al.’s idea in developing DeePromoter using non-promoters constructed from original promoters [45]. The idea was to introduce small fragments of functional motifs from promoters to non-promoters to overcome the model’s dependency on these motifs.

**Fig. 2:**
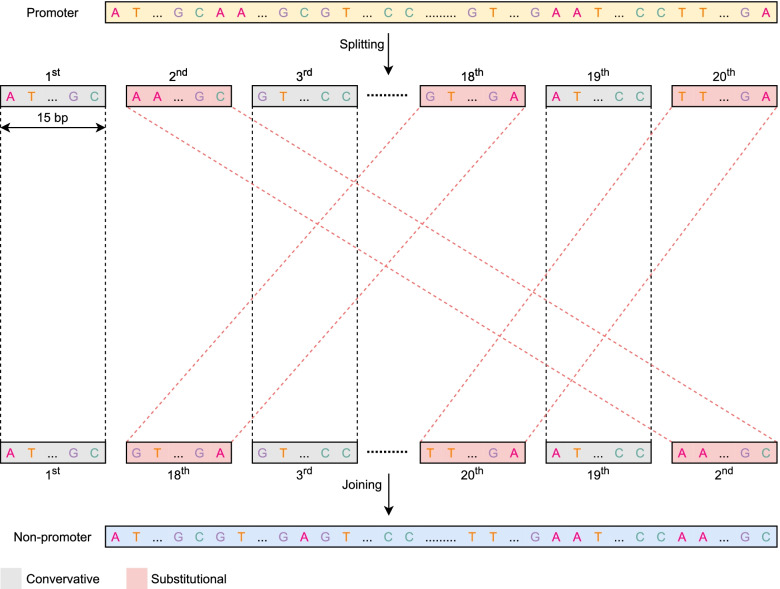
Construction of non-promoters (used in model training only) based on their corresponding promoters

Figure 2 describes key steps in constructing non-promoter sequences based on their corresponding promoter sequences. For each promoter sequence, we constructed a non-promoter sequence by recombination of some promoter subsequences while keeping other promoter subsequences at their original positions. Promoter subsequences having their positions unchanged are termed ‘conservative’. Promoter subsequences having their original positions interchanged by another one are termed ‘substitutional’ subsequences. Initially, promoter sequences of 300bp were equally split into 20 subsequences of 15bp. For each promoter sequence, 8 in 20 subsequences were randomly selected for recombination while the rest were kept immobile. The picked substitutional subsequences were then randomly filled in the gap positions until no gap remained. Finally, a new recombinant sequence was generated by joining all subsequences. Those artificial sequences which share minor structural similarities compared to corresponding promoter sequences were treated as non-promoter sequences for model training only. For each present promoter sequence, a corresponding artificial non-promoter sequence was created so that the ratios of promoters to artificial non-promoters in all datasets were equal (Table 2).

### Sequence-embedded features

**Fig. 3:**
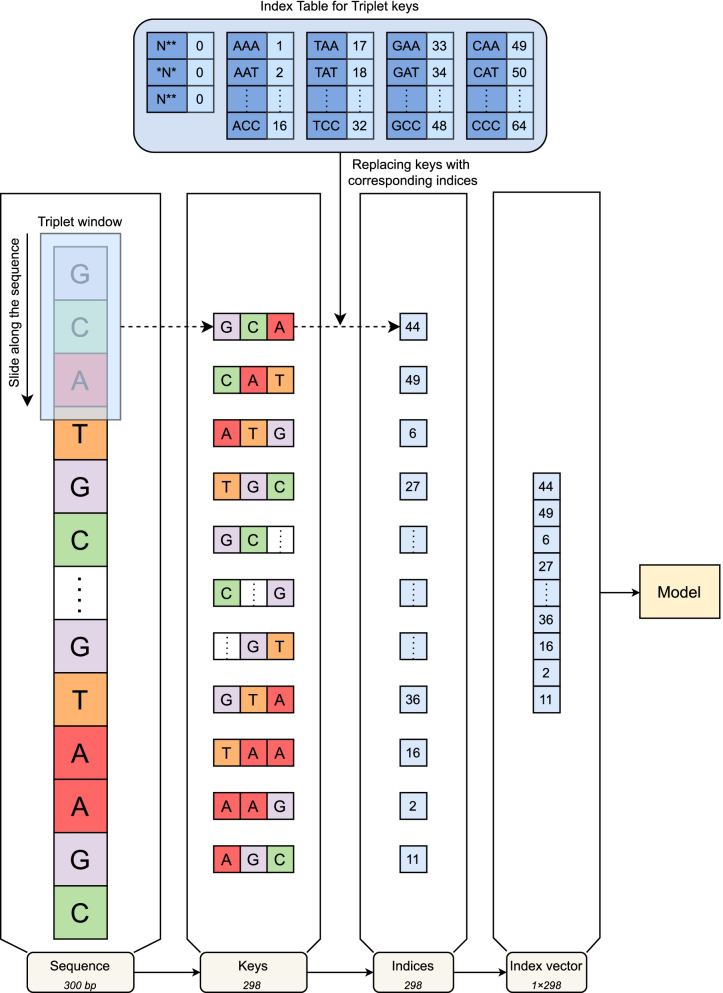
Conversion of sequences to index vectors

Figure 3 summarizes the steps involved in constructing index vectors for sequence samples in our study. Initially, an index table for triplet keys was created to store indices of triplet sets of consecutive nucleic acids. For a sequence, a window of 3 was used to read the whole sequence, starting at the first nucleic acid and terminating when reaching the final one. Since the sequence length is 300bp, the maximum number of triplet keys extracted is 298. Each triplet key was then looked up with the index table to get its corresponding index. Subsequently, a list of indices was obtained with a specific order and then joined to create an index vector of 1$\times$298. The index vectors were inputs of our models.

### Model architecture

**Fig. 4:**
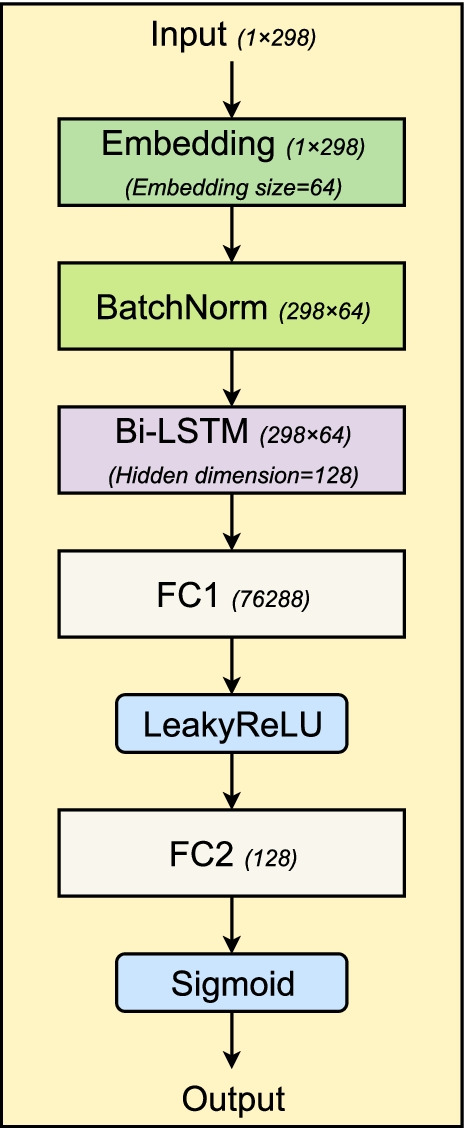
Model architecture

Figure 4 describes the model architecture designed to identify human TATA promoters, human non-TATA promoters, mice’s TATA promoters, and mice’s non-TATA promoters. The input data of the models are index vectors sized 1$\times$298. The input data first enters the embedding layer with an embedding size of 64 to create embedding matrices sized 298$\times$64 before passing through the batch normalization (BatchNorm) layer. The embedding layer receives data in the form of index vectors storing a series of indices. These indices come from the triplet sets of consecutive nucleic acids. The normalized matrices are the inputs of bidirectional long short-term memory (Bi-LSTM) layers designed with a hidden dimension of 128. Bi-LSTM activates a process of reading sequence information in both directions: forward and backward. Unlike regular LSTM models that use only one stream of input data, the Bi-LSTM model receives input streams in both directions. The Bi-LSTM layers transform normalized matrices sized 298$\times$64 to matrices sized 298$\times$256. These matrices are then flattened and passed through the first fully connected (FC1) layer activated by a Leaky Rectified Linear Unit (Leaky ReLU). After passing layer FC1, vectors sized 1$\times$76288 are converted to vectors sized 1$\times$128 which are gone through layer FC2 and finally activated by the sigmoid function to return probabilities. The loss function used is the binary cross-entropy which is expressed as:

1 $$\begin{aligned} Loss = \sum\limits _{i=1}^{n} y_i \times log\hat{y}_i + (1-y_i) \times log(1 -\hat{y}_i), \end{aligned}$$

where *y* is the true label and $\hat{y}$ is the predicted probability. The prediction threshold was set at 0.5 by default. The validation sets were used to define the stopping epochs for four models. For each model, the stopping epoch was the epoch where the validation loss was minimum. The Adam optimization algorithm [69] was used along with each minibatch of 64 samples. In our experiments, iPromoter-Seqvec was implemented using PyTorch 1.3.1 and trained on Google Colab equipped with 25 GB of RAM and one NVIDIA Tesla T4 GPU. iPromoter-Seqvec was trained over 50 epochs. It took about 15 seconds and 60 seconds to complete one training epoch for models predicting TATA promoters and models predicting non-TATA promoters, respectively. iPromoter-Seqvec requires 0.5 seconds and 3 seconds to complete testing models that predict TATA promoters and models that predict non-TATA promoters, respectively.

### Evaluation metrics

To assess the model performance, several metrics including balanced accuracy (BA), sensitivity (SN), specificity (SP), precision (PR), F1 score, Matthews’s correlation coefficient (MCC), the area under the receiver operating characteristic curve (AUCROC), and the area under the precision-recall curve (AUCPR) were measured. TP, FP, TN, and FN are abbreviated for True Positives, False Positives, True Negatives, and False Negatives, respectively. The mathematical formulas of these metrics are expressed below.

2 $$\begin{aligned} BA = \frac{SN + SP}{2} \end{aligned}$$

3 $$\begin{aligned} SN = \frac{TP}{TP + FN} \end{aligned}$$

4 $$\begin{aligned} SP = \frac{TN}{TN + FP} \end{aligned}$$

5 $$\begin{aligned} PR = \frac{TP}{{TP + FP}} \end{aligned}$$

6 $$\begin{aligned} F_1 = 2 \times \frac{{PR \times SN}}{{PR + SN}} \end{aligned}$$

7 $$\begin{aligned} MCC = \frac{TP \times TN - FP \times FN}{\sqrt{(TP + FP)(TP + FN)(TN + FP)(TN + FN)}} \end{aligned}$$

### Software availability

To support research communities to identify promoters, we deployed iPromoter-Seqvec as a user-friendly interface web server which can be accessed via https://github.com/mldlproject/2022-iPromoter-Seqvec. iPromoter-Seqvec supports identifying TATA and non-TATA promoters in human and mouse genomes. Users can follow simple steps described on the web server to perform their predictions task with iPromoter-Seqvec.

## Supplementary Information

**Additional file 1:** **Supplementary Table 1**: Model performance of iPromoter-Seqvec and DeePromoter on the validation sets. **Supplementary Figure 1**: ROC curves of iPromoter-Seqvec and iPro-EL on different independent test sets. **Supplementary Figure 2**: PR curves of iPromoter-Seqvec and iPro-EL on different independent test sets.

## Data Availability

To create the benchmark dataset, original data were collected from the Eukaryotic Promoter database [65, 66] and then independently refined. The benchmark datasets can be downloaded from our project website at https://github.com/mldlproject/2022-iPromoter-Seqvec. A web server implementing the proposed method can be accessed from there as well.

## Abbreviations

DNA: Deoxyribonucleic Acid
TSS: transcription start site
MCC: Matthew’s Correlation Coefficient
ROC: Receiver Operating Characteristic
PR: Precision-Recall
AUCROC: Area under the ROC curve
AUCPR: Area under the PR curve
