# Supplementary material for: iPromoter-Seqvec: identifying promoters using bidirectional long short-term memory and sequence-embedded features
Source: BMC Genomics. 2022 Oct 3;23(Suppl 5):681. doi: 10.1186/s12864-022-08829-6 (PMC9531353; doi:10.1186/s12864-022-08829-6)
Supplement: Supplementary file 1 — Additional file 1: Supplementary Table 1: Model performance of iPromoter-Seqvec and DeePromoter on the validation sets. Supplementary Figure 1: ROC curves of iPromoter-Seqvec and iPro-EL on different independent test sets. Supplementary Figure 2: PR curves of iPromoter-Seqvec and iPro-EL on different independent test sets. [file 12864_2022_8829_MOESM1_ESM.pdf]

Supplementary Information: iPromoter-Seqvec: Identifying Promoters using Bidirectional Long Short-Term Memory and Sequence-embedded Features

Thanh-Hoang Nguyen Vo,[†] Quang H. Trinh,[‡] Loc Nguyen,[†] Phuong-Uyen Nguyen-Hoang,[¶] Susanto Rahardja,[*,§,‖] and Binh P. Nguyen[*,†]

†*School of Mathematics and Statistics, Victoria University of Wellington, Wellington 6140, New Zealand*

‡*School of Information and Communication Technology, Hanoi University of Science and Technology, Hanoi 100000, Vietnam*

¶*Computational Biology Center, International University - VNU HCMC, Ho Chi Minh City 700000, Vietnam*

§*School of Marine Science and Technology, Northwestern Polytechnical University, Xi'an 710072, China*

‖*Infocomm Technology Cluster, Singapore Institute of Technology, Singapore 138683, Singapore*

E-mail: susantorahardja@ieee.org; binh.p.nguyen@vuw.ac.nz

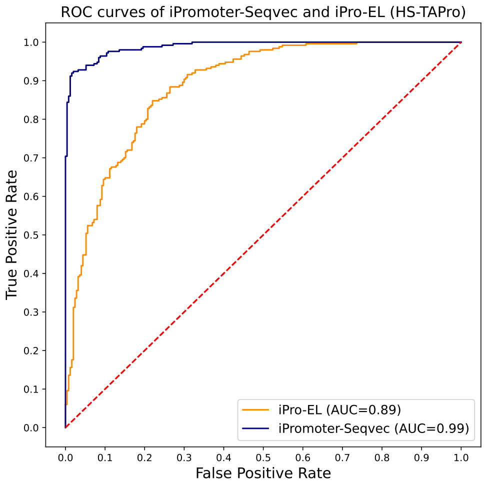

(a) HS-TAPro

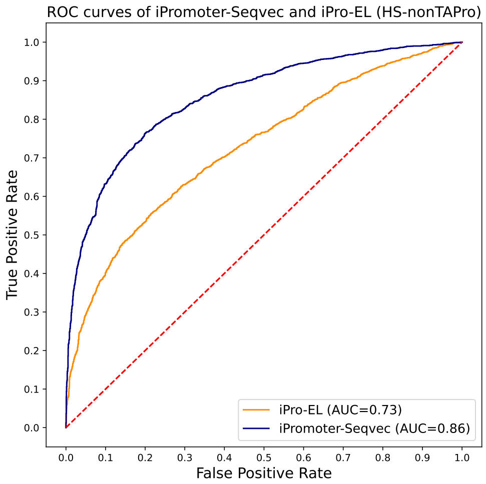

(b) HS-nonTAPro

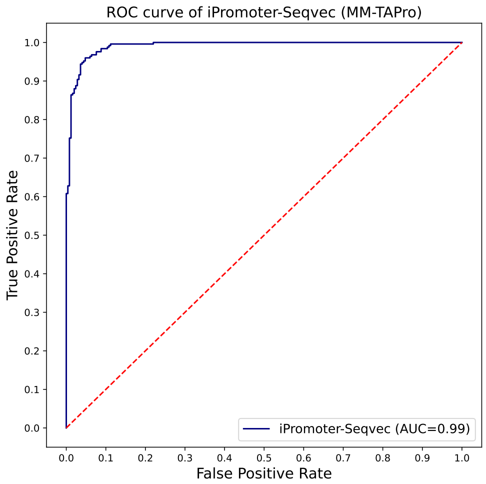

(c) MM-TAPro

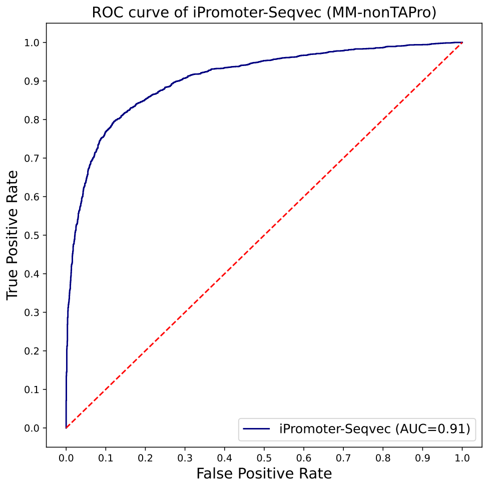

(d) MM-nonTAPro

Figure S1: ROC curves of iPromoter-Seqvec and iPro-EL on different independent test sets.

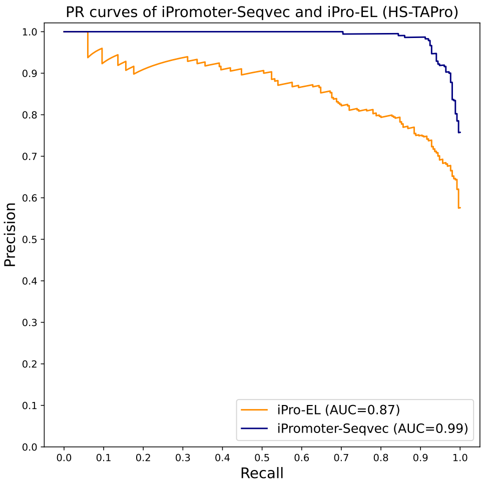

(a) HS-TAPro

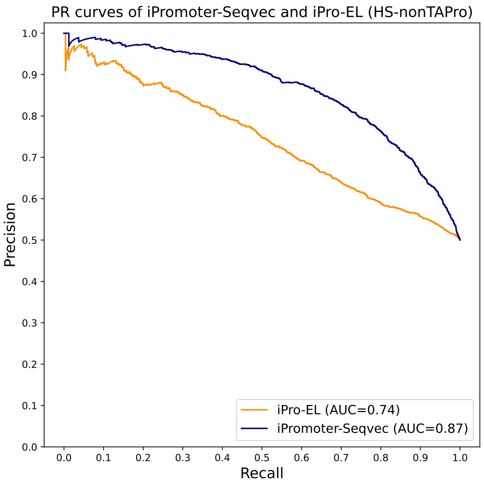

(b) HS-nonTAPro

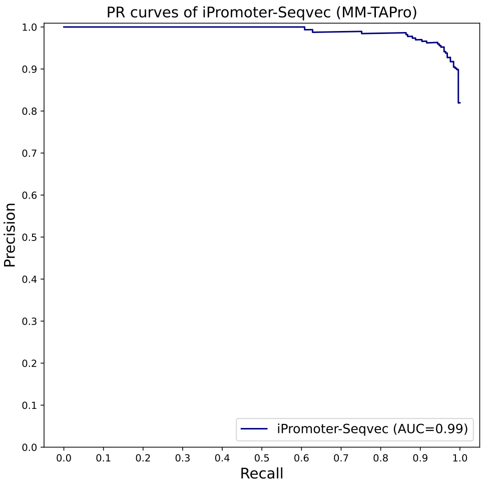

(c) MM-TAPro

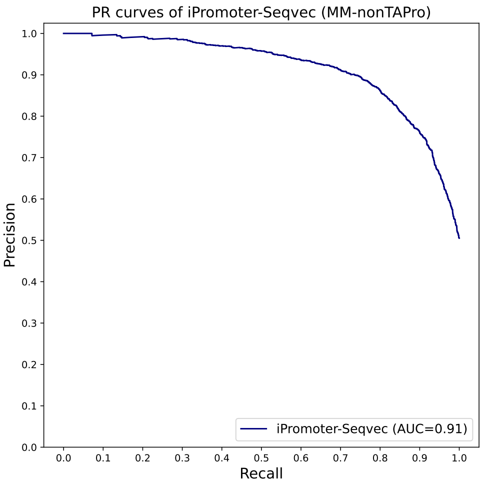

(d) MM-nonTAPro

Figure S2: PR curves of iPromoter-Seqvec and iPro-EL on different independent test sets.

Table S1: Model performance of iPromoter-Seqvec and DeePromoter on the validation sets.

**Dataset**	**Method**	**AUCROC**	**AUCPR**	**BA**	**SN**	**SP**	**PR**	**MCC**	**F1**
HS-TApro	DeePromoter	0.98	0.97	0.93	0.97	0.90	0.90	0.87	0.93
	iPromoter-Seqvec	0.98	0.98	0.92	0.89	0.96	0.95	0.85	0.92
HS-nonTApro	DeePromoter	0.68	0.68	0.64	0.55	0.72	0.66	0.28	0.60
	iPromoter-Seqvec	0.85	0.86	0.76	0.63	0.89	0.85	0.53	0.72
MM-TApro	DeePromoter	0.98	0.98	0.93	0.92	0.94	0.94	0.86	0.93
	iPromoter-Seqvec	0.99	0.99	0.93	0.89	0.98	0.98	0.87	0.93
MM-nonTApro	DeePromoter	0.75	0.76	0.69	0.65	0.72	0.70	0.38	0.68
	iPromoter-Seqvec	0.90	0.91	0.81	0.71	0.90	0.88	0.63	0.79
